# Alternatives to default shrinkage methods can improve prediction accuracy, calibration, and coverage: A methods comparison study

**DOI:** 10.1177/09622802251338440

**Published:** 2025-05-29

**Authors:** Mark A van de Wiel, Gwenaël GR Leday, Martijn W Heymans, Erik W van Zwet, Ailko H Zwinderman, Jeroen Hoogland

**Affiliations:** 1 Department of Epidemiology and Data Science, Amsterdam Public Health Research Institute, Amsterdam University Medical Centers, Amsterdam, the Netherlands; 2 Biometris, Wageningen University and Research, Wageningen, the Netherlands; 3 Department of Biomedical Data Sciences, Leiden University Medical Center, Leiden, the Netherlands

**Keywords:** Shrinkage, prediction, regression, calibration, coverage

## Abstract

While shrinkage is essential in high-dimensional settings, its use for low-dimensional regression-based prediction has been debated. It reduces variance, often leading to improved prediction accuracy. However, it also inevitably introduces bias, which may harm two other measures of predictive performance: calibration and coverage of confidence intervals. Here, the latter evaluates whether the amount of uncertainty is correctly quantified. Much of the criticism stems from the usage of standard shrinkage methods, such as lasso and ridge with a single, cross-validated penalty. Our aim is to show that readily available alternatives may improve predictive performance, in terms of accuracy, calibration or coverage. We study linear and logistic regression. For linear regression, we use small sample splits of a large, fairly typical epidemiological data set to illustrate that usage of differential ridge penalties for covariate groups may enhance prediction accuracy, while calibration and coverage benefit from additional shrinkage of the penalties. Bayesian hierarchical modeling facilitates the latter, including local shrinkage. In the logistic regression setting, we apply an external simulation to illustrate that local shrinkage may improve calibration with respect to global shrinkage, while providing better prediction accuracy than other solutions, like Firth’s correction. The potential benefits of the alternative shrinkage methods are easily accessible via example implementations in R, including the estimation of multiple penalties. A synthetic copy of the large data set is shared for reproducibility.

## 1. Introduction

Shrinkage has become a standard technique in statistics to counter over-fitting in regression models. In high-dimensional settings, with the number of variables p larger than sample size n , application of shrinkage is necessary to obtain parameter estimates and predictions. In low-dimensional settings, the benefit of shrinkage depends on the n:p ratio, as well as the main purpose of the analysis, for example, prediction, parameter estimation, variable selection, or causal effect estimation. Here, we focus on prediction. In general, the consensus is that, on average, shrinkage improves prediction accuracy on test data from the same population as the training data.^1–4^ This is no surprise as the penalty parameter is usually tuned, for example, by cross-validation, to maximize out-of-bag prediction accuracy. Hence, it will adapt to the n:p ratio: if large, little shrinkage is necessary; if small, more shrinkage is required. The use of shrinkage, however, comes at a price. It inevitably biases the parameter estimates, which in turn may lead to bad calibration of the prediction.^1,2^ Moreover, the penalty parameter can be rather instable,^2,3^ and such instability is often not communicated when presenting resulting models. Most empirical results that support these critiques on shrinkage are based on standard penalization techniques like lasso and ridge. While these issues are to some extent intrinsic to any shrinkage method, we show that they can sometimes be substantially alleviated by using alternatives that are only slightly more advanced. Of note, we do not aim to make a universal statement on the superiority of these methods as compared to standard ones, as this is likely not the case. Instead, our aim is to explain and illustrate to a broad audience of applied statisticians that “non-standard” shrinkage methods, such as group-adaptive and local shrinkage, can be very useful alternatives to standard ones for fitting multivariable prognostic models in epidemiological studies. For that we compare those methods with standard ones, such as ridge and ordinary least squares (OLS), on predictive accuracy, calibration and stability. As such this study complements the review by Friedrich et al.,^4^ which discusses several regularization techniques, including Bayesian ones, and their use in clinical biostatistics settings.

In terms of shrinkage, we focus on several variants of linear and logistic ridge regression. We do briefly discuss comparison of prediction accuracy with classical lasso and stepwise selection (which could be regarded as an extremely bimodal type of shrinkage) for the main data example, showing that these two are inferior to the ridge variations. We emphasize that lasso and stepwise selection cater mainly for variable selection, rendering the comparison with ridge-type models somewhat unfair. Therefore, we focus on the latter.

In regression, shrinkage is either effectuated by using a penalty for the parameters or an informative prior on these parameters in a Bayesian setting. In the latter case, the scale parameter of the prior distribution acts effectively as a penalty parameter, as the mean of the prior is usually set to zero. We explore group-adaptive shrinkage by the use of different penalties for (groups of) variables. We argue that in many settings it is in fact unreasonable to assume the same penalty for all variables. We show that both prediction accuracy and calibration may be greatly enhanced by using differential penalties. In addition, the use of differential penalties provides a more objective solution to down-weighting a set of variables than simply discarding it prior to the analysis, as is sometimes suggested as a (partial) solution for a low n:p ratio. Importantly, these penalties can be estimated automatically without (time-consuming) cross-validation in both the classical and full Bayesian setting, using R-packages mgcv,^5^ and R-Stan,^6^ respectively.

Global shrinkage methods, such as ridge, penalize all parameters equally. While such shrinkage likely improves predictive accuracy, calibration is often inferior to methods employing little to no shrinkage, as the reduced variability is countered by increased bias. While this is partly an inevitable consequence of shrinkage, we investigate whether Bayesian local shrinkage,^7–9^ which facilitates less shrinkage on strong variables, improves calibration.

As the penalty parameter(s) may be quite unstable^2,3^ it is important to propagate such instability towards the uncertainty of the predictions. Note here that instability in the very high ranges of the penalty parameters may be less relevant for predictions — as the regression parameters are strongly shrunken to zero anyway — than instability in the lower ranges. Therefore, penalty parameter variability is relevant, but not so easy to interpret. Instead, for a data set at hand, confidence or credible intervals for the predictions may be easier to interpret (as these are on the same scale as the outcome) *if* these intervals indeed have the desired coverage (say 95%) of the true values. By comparing coverage, we evaluate which methods correctly quantify the amount of uncertainty, including that of the penalty parameter(s). Note that the Bayesian shrinkage models naturally account for the penalty parameter uncertainty, and their hierarchical shrinkage may provide improved stability of the penalty parameters. In the frequentist paradigm, penalty parameter uncertainty is not explicitly modeled. Marra and Wood^10^ show, however, that modifying the classical intervals such that the covariance matrix of the regression coefficients matches with the Bayesian one improves coverage. Here, we also consider those “unconditional” intervals, as implemented in mgcv.

For the linear regression setting, our main strategy to compare methods is to divide a large ( N=21,570 ) study into many subsets of sizes n=100 and n=200 . The choice for these sample sizes is a pragmatic one: while often we, and other statisticians, recommend to use larger ones, this is not always feasible in practice, at least not during the initial stages of the prediction model development. To study to what extent differences between shrinkage methods diminish with increasing sample size, we also provide comparisons for n=320 . The latter results from a sample size calculation,^11^ which is based on reducing the need for shrinkage by lower bounding a global shrinkage factor. We use 12 variables to predict the outcome and add five random noise covariates to make sure that some covariates are not linked to the outcome at all, resulting in p=17 . The OLS results on the entire set are used as a benchmark, as these estimates are unbiased and very precise due to the large sample size. Then, the subsets are analyzed with various shrinkage methods and compared to the benchmark to evaluate prediction accuracy (using out-of-bag test samples), calibration and coverage of the prediction interval. For the logistic regression setting, we also evaluate predictive accuracy, but we focus on calibration, as this was shown to be a major concern in the case of binary outcome when using standard global shrinkage approaches.^1^ We align with their simulation setting to demonstrate that Bayesian local shrinkage is a better alternative, highly competitive to mild shrinkage strategies like using a fixed ridge penalty^12^ or Firth’s correction.^13^

### 1.1. Data

For the linear regression setting, the main data we use throughout the manuscript is obtained from the Helius study.^14^ We study response Y : systolic blood pressure (SBP), as a function of covariates X : age, gender, BMI, ethnicity (five levels), smoking (binary), packyears, coffee (binary), glucose (log), cholesterol, and rendering 12 covariates after dummy coding the nominal covariate. We apply minimal preprocessing to the data: only 2.7% of the samples had at least one missing value; these samples were removed, rendering a sample size N=21,570 . Continuous covariates were standardized, as this is common practice before applying shrinkage for the penalty to have the same effect on all corresponding regressing parameters. For binary covariates, the standardization itself may become unstable for small data sets, in particular when the classes are unbalanced. This may hamper generalization to test settings. Therefore, we opted for a default − 1, 1 coding, as this standardizes a balanced binary covariate. For our data, we compared results with those from complete standardization. Differences are small, but marginally better for the proposed coding. Empirical correlations between covariates are displayed in Supplemental Table 1. Absolute correlations range from 0 to 0.51, with 13/66 absolute correlations larger than 0.2. Finally, we added five independent standard normal noise covariates. Hereby, we are sure to include some covariates completely unrelated to the outcome, SBP. Hence, the total number of covariates equals 17.

We chose this data set for various reasons. First, it addresses a fairly standard, and well-known prediction problem with an interesting mix of covariates (binary, nominal and continuous). Second, its large sample size allows to (a) use the OLS estimates from the entire set as the benchmark, because these have very small standard errors (typically ≈0.01 ); and (b) to split the set in many independent subsets with sample sizes as often encountered in clinical studies. This enables us to evaluate various (shrinkage) methods on many real, relatively small sample data sets. Third, from applying OLS to the entire data set, we have $R^{2}=0.34$ , which is neither trivially low nor high.

For the logistic regression setting, we use an external simulation to concur with existing studies. The set-up is described further on.

## 2. Methods

We introduce the methods in the context of the linear model: $Y_{i}=\beta_{0}+X_{i}\beta =\beta_{0}+\sum_{j=1}^{17}\beta_{j}X_{ij}+\epsilon_{i},\epsilon_{i}∼N(0,\sigma^{2})$ , with i=1,…,n , $\beta =(\beta_{1},\ldots ,\beta_{17})$ and covariates $X_{i}=(X_{i1},\ldots ,X_{i17})$ . The benchmark value of β , ${\hat{\beta }}^{\text{L}}$ , is obtained by applying OLS to the entire data set (of size N ) which rendered estimates with extremely small standard errors. This does not imply that we believe the linear model to be the “best” model. In fact, for such a large N , alternatives that allow for interactions or non-linear effects may be superior. However, we should emphasize that our focus lies on small data sets with a fairly large number of covariates, a setting for which fitting more complex models is likely futile. Moreover, residual plots based on the entire data set do not show strong deviations from either linearity or homoscedasticity (Supplemental Figures 1 and 2). Therefore, it is reasonable to use the OLS results of the entire data set as a benchmark. As the small training sets are orders of magnitude smaller than the entire set ( N/n>100 ), each of these has negligible impact on the benchmark, which we, therefore, keep fixed. We first discuss the methods for fitting the model. Note that part of the methods is also applied to the logistic regression setting, sometimes with small adjustments. Details are provided further on.

### 2.1. Standard solutions

We study OLS, stepwise selection (“step”), lasso and ridge as standard solutions with no or just one global penalty parameter. The intercept is not penalized. Note that step is also a shrinkage method, but of an extreme nature: it shrinks completely to 0 or not at all, hence somewhat similar to the use of a spike-and-slab prior in a Bayesian model. Here, step is the standard step implementation in R, using forward-backward selection and AIC to select a model. We use lasso as implemented in glmnet, using lambda.min, the value of the penalty parameter that minimizes the error estimated by 10-fold cross-validation (CV). For ridge, we estimated penalties using marginal likelihood optimization as implemented in mgcv,^5^ which, as opposed to glmnet, provides confidence intervals.^10^ Results from 10-fold CV were very similar, and hence not shown.

### 2.2. Multi-penalty solutions: Group-adaptive shrinkage

Multi-penalty solutions allow the use of different penalties for groups of covariates, that is, group-adaptive shrinkage. Groups of interest include those for which prior knowledge suggests that differential shrinkage is required and those that follow from modeling choices that induce groups of parameters (e.g. for categorical variables). We focus on ridge-type solutions for three reasons: (a) our focus lies on prediction and not on variable selection; (b) standard software implementations such as mgcv allow efficient estimation of the penalties by means of maximizing the marginal likelihood^5^; and (c) standard ridge performs relatively well. In some settings, the use of covariate groups is very natural, for example when some covariates represent similar entities (e.g. genes), or when main effects plus interactions are included. In other cases, like ours, choice of the covariate groups implies a level of subjectivity. Therefore, we also assess the performance of multi-penalty approaches when the covariate groups are chosen randomly; that is, when the prior information used to form the covariate groups is useless. Besides differential penalization another option is not to shrink one (group of) covariates, for example, because there is substantial evidence that these effect the outcome. We will explore this option as well. We now define the covariate groups that were used:

- Two groups, G=2 . The first three covariates (age, gender, and BMI) are one group, because these are known to relate to the outcome, SBP, from previous studies. The other 14 covariates are in the second group. Two options are explored: the method denoted by ridge_2 penalizes both groups, separately, whereas the method ridge_2un leaves the first group unpenalized. This type of covariate grouping reflects a common case: one group of covariates for which there is strong evidence of association with the outcome from previous studies, and one group of covariates with either weak or absent evidence.
- Three groups, G=3 ; method denoted by ridge_3. As above, but the nominal covariate, ethnicity, is a seperate group as this is a nominal covariate with five levels; here, “Dutch” is chosen as the baseline. The third group illustrates another common covariate grouping setting: the dummies that represent the levels of a multi-level nominal covariate.
- Two random groups. Three covariates are randomly picked to belong to group 1, the other 14 belong to group 2. Randomization is repeated for each training data set. Referred to as ridge_2r and ridge_2unr, the latter corresponding to leaving the smallest group of covariates unpenalized.

The penalty parameters for standard solutions like ridge may be rather unstable.^2,3^ This instability is likely to increase when estimating multiple penalty parameters, as less data per penalty parameter is available then. Bayesian solutions may be a worthwhile alternative to shrink these penalties with an additional shrinkage prior to counter such instability. Moreover, the extreme case: one penalty per parameter, referred to as local shrinkage, may benefit calibration, as illustrated further on.

### 2.3. Bayesian solutions

Shrinkage is a very natural concept in Bayesian statistics. Depending on the type of shrinkage, frequentist and Bayesian solutions may be very similar. For example, the frequentist ridge and lasso estimates of β are equal to the posterior mode estimate of β when using a Gaussian or Laplacian prior with a fixed precision parameter that relates proportionally to the penalty parameter for fixed sample size.

Bayesian methodology, however, can hierarchically model the variability of the penalty parameter, which holds several promises. First, it counters potential instability of estimates of penalty parameter(s) by using a (weakly) informative prior for it. Second, it propagates uncertainty of penalty parameter(s). This follows from the fact that for a single β and random penalty parameter λ we have for its variance: $V(\beta )=E_{\lambda }[V(\beta |\lambda )]+V_{\lambda }[E(\beta |\lambda )].$ Finally, it allows local shrinkage, either by group, or even per covariate. Note that, just as for OLS, an advantage of local shrinkage is that the results are less sensitive to (differences in) scale and effect sizes, because the local penalty is able to adapt.

**Figure 1.**
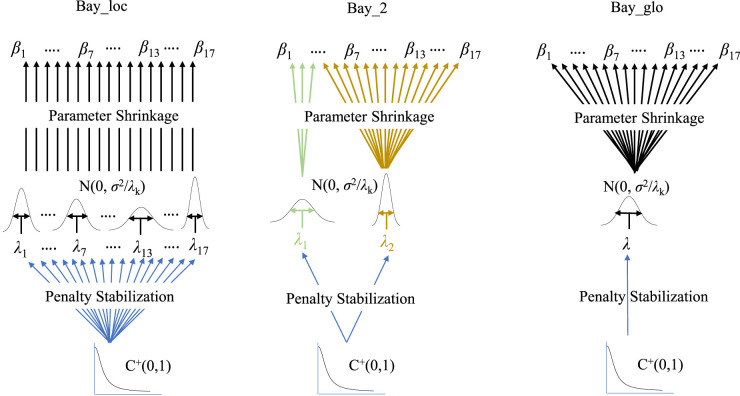
Illustration of priors in Table 1: local (Bay_loc), group-adaptive (Bay_2), and global (Bay_glo).

Extensions to Bayesian ridge regression differ in how they allow the penalty parameter(s) to vary. We focus on the half-Cauchy prior, $C^{+}(0,1)$ , for the standard deviation ( $=\lambda^{-1/2}$ ) which has fairly heavy tails and was advocated as a good default prior by Gelman^7^ and Polson and Scott.^8^ Table 1 specifies the local, group-adaptive and global penalization models. The hierarchical nature of these models, and how they potentially stabilize the penalties, is illustrated in Figure 1. Moreover, $\sigma^{2}$ , the error variance in the linear model (set to 1 in above’s prior for the logistic), is endowed with a default, vague inverse gamma prior. It is standard practice to include $\sigma^{2}$ in the prior of $\beta_{k}$ in Bayesian linear regression. We have also compared two other standard (empirical) Bayesian ridge regression solutions with their frequentist counterpart, ridge. The first one uses a fixed empirical Bayes estimate of λ , whereas the other applies the commonly used vague gamma prior on precision parameter λ . Supplemental Figure 5 shows very little difference in predictive performance between those methods as compared to ridge. This is expected as for both methods the posterior mode estimate coincides with the frequentist estimate. Given these small differences, we focus on the priors in Table 1 for further evaluations.

**Table 1.** Priors. Here, g(k) denotes group g∈{1,…,G} , with G=2,3 , to which variable k belongs.

Name	Prior β	Prior penalty λ	Type of shrinkage
Bay_loc	$\beta_{k}∼N(0,\sigma^{2}\lambda_{k}^{-1})$	$\lambda_{k}^{-1/2}∼{\text{C}}^{+}(0,1)$	Local
Bay_2 (3)	$\beta_{k}∼N(0,\sigma^{2}\lambda_{g(k))}^{-1}$	$\lambda_{g}^{-1/2}∼{\text{C}}^{+}(0,1)$	Group-adaptive
Bay_glo	$\beta_{k}∼N(0,\sigma^{2}\lambda^{-1})$	$\lambda^{-1/2}∼{\text{C}}^{+}(0,1)$	Global

### 2.4. Evaluation criteria

Training sets $T^{s}$ and their complementary test sets $T^{\prime s}$ are indexed by s . The benchmark value of the prediction for any (test) sample i with covariates $X_{i}$ then equals $\eta_{i}={\hat{\beta }}_{0}^{\text{L}}+X_{i}{\hat{\beta }}^{\text{L}}$ . We evaluate the methods on three criteria:

1. *Prediction accuracy*. We use the mean squared error of predictions (MSEp). For a given subset s , this is defined as follows:
(1) $${\text{MSEp}}^{s}=1/|T^{\prime s}|\sum_{i\in T^{\prime s}}(\eta_{i}-{\hat{\eta }}_{i}^{s}{)}^{2}$$
  with, for test sample i , ${\hat{\eta }}_{i}^{s}={\hat{\beta }}_{0}^{s}+X_{i}{\hat{\beta }}^{s}$ , where estimates ${\hat{\beta }}_{0}^{s}$ and ${\hat{\beta }}^{s}$ are obtained from samples in training set $T_{s}$ . As we have the benchmark predicted values $\eta_{i}$ in this setting, we use those in (1) instead of $y_{i}$ , which would render the prediction squared error. Of note: the latter deviates from the former by a constant (see Supplemental Section 1), so both lead to the same conclusions in terms of methods comparison.
2. *Calibration of the predictions*. We evaluate calibration by regressing the observed test set values $y_{i}^{s}$ on the predictions, ${\hat{\eta }}_{i}^{s}$ . A method calibrates well when the regression slope is close to 1.^1^
3. *Uncertainty quantification of the predictions*. We evaluate this by the mean coverage of the confidence intervals of the predictions $\eta =(\eta_{1},\ldots ,\eta_{N})$ . In the classical penalized ridge regression setting, we use the 95% Gaussian Wald intervals with standard errors corrected for prior uncertainty of β , as derived by Marra and Wood.^10^ For Bayesian methods, we simply use the (2.5% and 97.5%) quantiles of the posteriors to create 95% credible intervals for the predictions $\eta_{i}$ . A method quantifies uncertainty correctly when the coverage is close to 95%.

## 3. Results, linear case

We show results based on 400 training subsets of sizes n=100,200,320 , which due to the size of the entire data set show no or little overlap in samples. Here, n=320 is the minimum recommended sample size to limit the need for shrinkage^11^; see the Supplemental Material for details on the calculation.

### 3.1. Prediction accuracy

We first consider prediction accuracy, as measured by the mean squared error of the predictions, MSEp (1). Predictions are evaluated on complementary test sets. Below, we discuss the results for several comparisons.

**Standard solutions**: We first compare the standard methods. Supplemental Figure 4 depicts the performances. For n=100 , we observe that ridge performs better than lasso, which is substantially better than stepwise selection and OLS. It also shows that for n=200,320 the gap between ridge and lasso becomes much smaller, and so do the other gaps.

**Figure 2.**
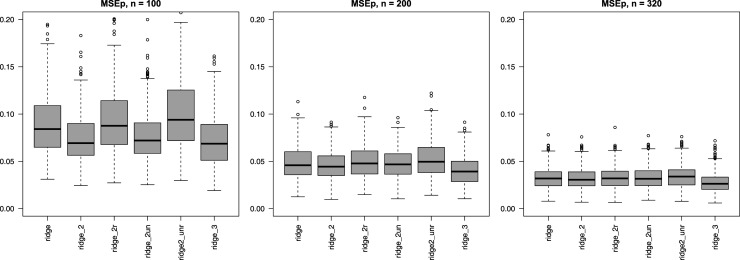
Mean squared error of the predictions (MSEp) (*y*-axis) for group-adaptive ridge penalization across 400 subsets. Digital suffix denotes number of covariate groups, suffix “un” denotes the one unpenalized covariate group, suffix “r” denotes the random covariate groups.

**Figure 3.**
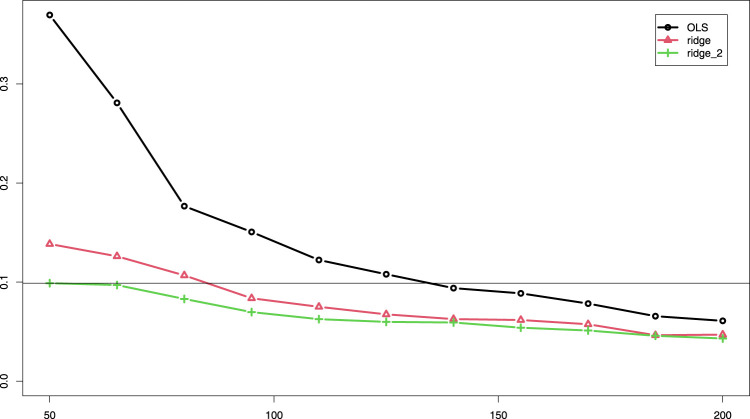
Median MSEp (*y*-axis) across 400 subsets for various sample sizes (*x*-axis) for OLS, ridge and two-group ridge. MSEp: mean squared error of the predictions; OLS: ordinary least squares.

**Multi-penalty solutions, group-adaptive shrinkage**: Figure 2 shows that use of group-adaptive shrinkage can improve prediction accuracy (lower MSEp). For n=100 , we observe an improvement for G=2 penalties, likely caused by the large difference in penalties for the two groups (see Supplemental Figure 7). Note that the relative reduction from ridge to ridge_2 of mean MSEp equals 16.0%. For n=200,320 , the improvement for G=2 is marginal, but the larger sample size benefits the estimation of one more penalty rendering improved accuracy for G=3 (e.g. relative reduction MSEp ridge to ridge_3 equals 15.9% for n=200 ). In this setting ridge_2un, which does not penalize the first three covariates, performs on par with ridge_2. This is reasonable, because these covariates are relatively strong, so need little penalization. In addition, the figure shows that when two groups are assigned at random ridge_2unr performs inferior to ridge_2r (with suffix “r” denoting random groups), also in terms of instability, as it cannot adapt the penalty for the relatively weak, unpenalized covariate group. Importantly, observe that even when the groups are random, ridge_2r performs — on average — on par with ridge, again due to the adaptive penalties. The improvement with the group-adaptive approach becomes very tangible in Figure 3: OLS and ridge need many more samples to achieve the same median MSEp as ridge_2, f.e. ∼ 135 and 85, respectively, versus 50, as indicated by the horizontal line.

**Figure 4.**
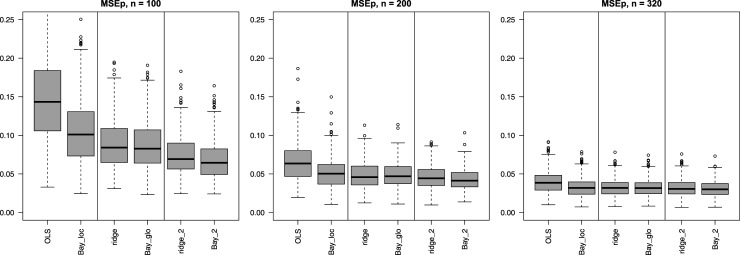
Mean squared error of the predictions (MSEp) (*y*-axis) for classical methods and their Bayesian counterpart across 400 subsets. Suffices “loc” and “glo” refer to local and global shrinkage, respectively (Figure 1). Digital suffix denotes the number of covariate groups for group-adaptive shrinkage.

**Adding Bayesian solutions**: Figure 4 compares each of the three Bayesian methods to their frequentist counterparts. Here, Bay_loc is contrasted with OLS as both methods do not imply any grouping on the covariates. Likewise, Bay_2 is contrasted with ridge_2 (two groups) and Bay_glo is contrasted with ridge. For the latter two comparisons we observe that differences in MSEp are minor. A more striking difference is observed for OLS vs Bay_loc, in particular for n=100 . While the latter cannot compete with the global and group-adaptive shrinkage methods (for n=100 ), it does perform much better than OLS, also in terms of stability. Apparently, the default shrinkage of the normal-C ${}^{+}$ prior substantially stabilizes the β estimates in such small sample size settings. The effect of the local normal-C ${}^{+}$ prior for β is depicted in Supplemental Figure 8 for two n=100 subsets with extreme OLS estimates of one of the most important coefficients, $\beta_{\text{BMI}}$ . We observe that the normal-C ${}^{+}$ prior of Bay_loc shrinks the extreme OLS estimates in the right direction. Naturally, methods perform more similarly when the sample size increases, as this diminishes the effect of shrinkage.

### 3.2. Calibration

**Figure 5.**
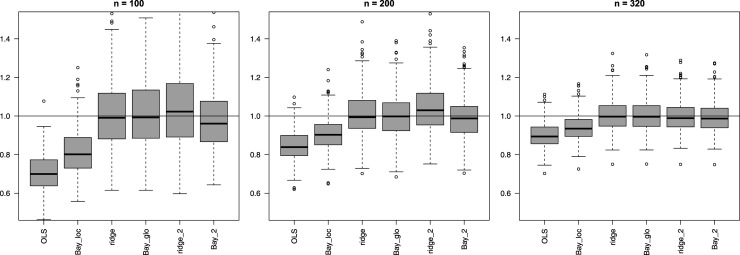
Calibration slopes (*y*-axis) across 400 subsets. Suffices “loc” and “glo” refer to local and global shrinkage, respectively (Figure 1). Digital suffix denotes the number of covariate groups for group-adaptive shrinkage.

Figure 5 shows the calibration slopes for n=100,200,320 and all subsets s for OLS, Bay_loc, ridge, Bay_glo, ridge_2, and Bay_2. Note that this slope is influenced by both bias and variance of the predictions.^15^ Therefore, methods that overfit likely render slopes smaller than 1, as the test set predictions are more extreme than the actual values. We observe this in Figure 5 for OLS. Bay_loc, which provides a bit of shrinkage, does better. The global shrinkage methods, ridge and Bay_glo, center better, but show a very large variability in slope for n=100 , confirming the results by Van Calster et al.^1^ Bayesian group-adaptive shrinkage (Bay_2) reduces this variability somewhat. Differences between methods become smaller with increasing sample size.

As the usual calibration slope quantifies a mix of bias and variance, we also regressed the test sample predictions ${\hat{\eta }}_{i}^{s}$ against the true benchmarks $\eta_{i}$ to focus more on the bias component. Supplemental Figure 9 shows the bias problem due to overshrinkage: generally, ridge and Bay_glo, and to a lesser extent ridge_2 and Bay_2, overshrink some coefficients, which compresses the test sample predictions as compared to the benchmark. As expected, OLS does not have this bias, and the local shrinkage by Bay_loc seems to largely prevent such bias as well.

To elaborate on the overshrinkage of ridge and Bay_glo compared to OLS and Bay_loc consider the estimated coefficients of a strong covariate (in the entire study), BMI, from the n=100 subsets. Supplemental Figure 10 clearly shows that the ridge and Bay_glo over-shrink $\beta_{\text{BMI}}$ due to the common shrinkage factor shared with the weaker covariates, whereas Bay_loc strikes a good balance between a small amount of bias and reducing variability compared to the OLS estimates.

### 3.3. Coverage of confidence intervals of predictions

Uncertainty of the predictions η results from the variability of β , which includes two components: one conditional on fixed penalty parameter(s) λ and one reflecting the variability of λ . The latter may be considerable^3^ (see also the Supplemental Material). The penalties are, however, usually not the primary estimands of interest; what matters most is to what extent such variability propagates towards β and, eventually, the predictions η . Should the variability of the penalties not appropriately be accounted for, this should lead to confidence intervals that do not have the targeted coverage. Hence, we finalize the comparison by evaluating coverages of the confidence intervals of η .

As before, we focus on OLS, ridge and ridge_2 and contrast these with Bay_loc, Bay_glo and Bay_2 in Table 2. Both ridge and ridge_2 are used with uncertainty propagation of the penalties, as implemented in mgcv’s predict.gam function with argument unconditional = TRUE. First, for n=100 , we observe that Bay_loc is competitive to OLS: while its coverage is somewhat higher than targeted, the resulting intervals are approximately of the same width. Second, ridge is somewhat less conservative than Bay_glo in terms of coverage, with slightly narrower intervals. Finally, for the group-adaptive penalties the extra shrinkage imposed by Bay_2 pays off as it maintains good coverage, unlike ridge_2, which seems to render too narrow intervals. For n=200,320 , the Bayesian procedures render somewhat more conservative intervals (higher coverage) than their classical counterparts, leading to somewhat wider intervals, but also to better coverage for the group-adaptive setting (cf. ridge_2 and Bay_2).

**Table 2.** Mean coverage (target: 0.95) and mean width of confidence intervals of predictions for n=100,200,320 using classical (Class) and Bayesian (Bay) methods. The mean is computed over 2000 test individuals, randomly selected. Suffices “loc” and “glo” refer to local and global shrinkage. Digital suffix denotes number of covariate groups for group-adaptive shrinkage.

	n=100				n=200				n=320			
	Coverage		Width		Coverage		Width		Coverage		Width	
Methods	Class	Bay	Class	Bay	Class	Bay	Class	Bay	Class	Bay	Class	Bay
OLS, Bay_loc	0.945	0.978	1.460	1.528	0.949	0.984	0.980	1.186	0.950	0.986	0.762	0.976
ridge, Bay_glo	0.950	0.967	1.160	1.281	0.953	0.973	0.864	1.000	0.952	0.976	0.701	0.844
ridge_2, Bay_2	0.883	0.954	0.940	1.101	0.905	0.952	0.746	0.870	0.921	0.957	0.634	0.759

### 3.4. Results for a small sample size setting, n=50

Supplemental Section 5 shows the results for n=50 . These are presented separately as one may argue such a sample size to be too small for developing a prediction model.^11^ Still, it serves as an extreme case that sometimes occurs in practice. Generally, the results are similar to those of n=100 , with differences between methods magnified.

### 3.5. Conclusions, linear case

Below we list the conclusions from the linear regression case.

1. Shrinkage benefits prediction accuracy compared to OLS and stepwise selection.
2. Group-adaptive shrinkage may benefit prediction accuracy compared to global shrinkage. Therefore, the same prediction accuracy can be achieved with a smaller sample size.
3. Bayesian local shrinkage outperforms OLS on both prediction accuracy and calibration, as it reduces variability at the cost of very little bias. The two are competitive in terms of coverage. Prediction accuracies are worse, though, than that of the global and group-adaptive shrinkage methods.
4. Bayesian group-adaptive shrinkage outperforms its frequentist counterparts, ridge, in terms of coverage, whereas the two are competitive in terms of prediction accuracy and calibration.
5. Differences in prediction accuracy and calibration between various (shrinkage) methods become smaller with increasing sample size.

## 4. Logistic regression: Calibration and predictive accuracy

Here, we study the logistic regression setting. This differs from the linear one in three essential ways: (a) binary outcomes are much less information-rich than continuous ones; (b) the logistic function flattens the impact of large β ’s; and (c) the maximum likelihood estimator (MLE) is biased.^13^ We focus on calibration, as this has been identified as a major concern in medical settings^16^ and standard global shrinkage methods with tuned parameters are not optimal for that purpose.^1^ This is intrinsic due to the introduced bias, but may be worsened by the conventional tuning of the penalty parameter: minimization of cross-validated prediction error, which may be far from optimal for calibration. Hence, we discuss some alternatives. Firth’s solution to adjust bias^13^ uses a fixed penalty, defined on the level of the information matrix, and was shown to perform favorably to the tuning methods.^1^ Therefore, the latter, such as ridge with cross-validation, are not considered here. Besides calibration we also evaluate predictive accuracy. We refrain from evaluating coverage of intervals, as these are likely very wide (hence uninformative) in the logistic regression setting when sample size is limited.

Sullivan and Greenland^12^ propose an alternative solution to Firth’s, which also accounts for the aforementioned issues (a) and (b): simply fix the prior variance of the β ’s to 0.5, equivalent to setting the ridge penalty λ=1/0.5=2 . Their argument, further supported by Sinkovec et al.,^2^ is that for the small sample logistic setting one has to “dare” to be fairly informative, as the outcomes are information-poor. Based on this, we propose a somewhat more objective alternative that does not require any tuning either: use Bay_loc as defined in Table 1, but with a $C^{+}(0,\sqrt{0.5})$ instead of a $C^{+}(0,1)$ prior for the standard deviation, as the former matches to a median prior variance of 0.5. These priors are depicted in Supplemental Figure 3. So, we allow for some variation in the prior variances, thereby enabling adaptation to cases in which (some of the) β ’s are more (or less) extreme.

**Figure 6.**
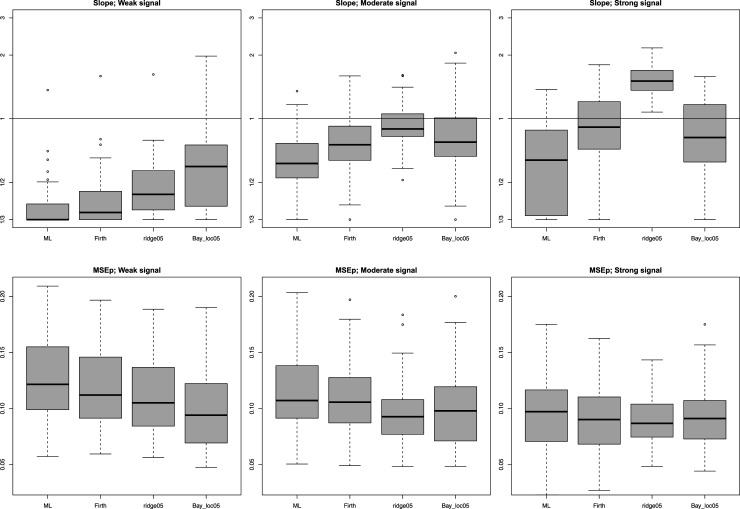
Calibration slope (top; winsorized at (1/3,3) , log-scale) and prediction accuracy (bottom; MSEp) for logistic regression simulations, n=100 . Suffix “05” refers to a prior variance or reciprocal ridge penalty equal to 0.5; Suffix “loc” refers to the local shrinkage (Figure 1).

For evaluating methods, we follow an external simulation set-up,^1^ which allows us to qualitatively compare with their results and to vary the strength of the signal. This set-up is: n=100 , $\beta =(\beta_{1},\ldots ,\beta_{5})=(0.2,0.2,0.2,0.5,0.8)$ and intercept $\beta_{0}$ tuned such that the average event probability equals 0.5, rendering a setting of 50/5 = 10 events per covariate. Covariates X are simulated from a multivariate normal with means 0, variances 1, and correlations 0.5. Response Y is then simulated from a Bernoulli with success probabilities $1/(1+\exp\;(-\beta_{0}-X\beta )).$ We refer to this setting as “Moderate signal.” Additionally, to study how results adapt to the signal strength, we simulate the “Weak and Strong signal” settings by using $\beta_{\text{weak}}=\beta /3$ , and $\beta_{\text{strong}}=3\beta$ . Calibration is assessed by the calibration slope. The MSE of the predictions (MSEp) is assessed by the average squared error of those predicted probabilities w.r.t. the true ones. Figure 6 displays the results for n=100 based on 50 training sets for ML (maximum likelihood); Firth; ridge05: ridge with fixed penalty 1/0.5; and Bay_loc05: local regularization with $C^{+}(0,\sqrt{0.5})$ priors for the standard deviation(s). Supplemental Figure 14 shows the results for n=500 . Below we list the overall conclusions.

- We confirm that Firth calibrates better than ML.^1^ It also improves prediction slightly.
- The solution ridge05^12^ is competitive to Firth in terms of calibration when the signal is weak or moderate, but overshrinking when the signal is strong. It is generally somewhat better than Firth for prediction.
- Bay_loc05 calibrates and predicts better than Firth in the weak signal setting and is competitive on both performance measures in the two other settings.
- Differences become smaller for increasing sample size.^1^

Supplemental Figure 15 presents the results for n=100 for an alternative scenario (adding five β ’s equaling 0). Most results are qualitatively similar to those presented here, but with somewhat stronger differences between methods. Hence, Bay_loc05 is a good alternative to Firth and ridge05. Moreover, unlike Firth and ridge05, it straightforwardly provides uncertainty estimation of the predictions, as these are automatically available from the credible intervals. As mentioned before, the calibration slope is influenced by both bias and variance of the predictions.^15^ Moreover, it is a global metric. Therefore, we assessed bias more locally by calibration curves.^17^ Supplemental Figure 16 shows these for n=100,500 and the moderate signal scenario. We clearly observe that increase of sample size decreases the variability between those curves. Differences between methods are minor, though, probably because all methods employ limited shrinkage.

## 5. Software and reproducibility

### 5.1. Software

Below, we list the software packages used for the various models.

1. OLS and stepwise selection: R’s lm and step functions.
2. Linear ridge, global and group-adaptive penalty: gam function in R-package mgcv.^5^ Unlike glmnet this allows for uncertainty computations.
3. Logistic ridge: glmnet, as this was also used by Van Calster et al.^1^ Firth’s correction: logistf.^18^
4. Lasso: glmnet.^19^
5. Bayes, linear: shrinkage software,^20^ which is optimized for computational efficiency, hence convenient for the repeated calculations on data splits. Results highly agree with those of r-stan.
6. Bayes, logistic: r-stan.^6^

### 5.2. Data sharing

As is the case for many cohort studies, our primary data source, the Helius data, cannot be shared publicly. This is impeding methodological studies like this, because such large N studies are very useful to check results of various methods on small subsets as we did. Therefore, we share a synthetic copy of our data set which may be used to (a) qualitatively reproduce our results; or (b) evaluate other methods than those proposed here. We verified whether results from the synthetic copy agreed with those from the real data, which is the case (Supplemental Figure 17 depicts the MSEp). The imputation-based method to generate the synthetic data is described in the Supplemental Material.

### 5.3. Availability

Annotated software scripts and R-markdown files to reproduce our results are available from github: https://github.com/markvdwiel/ThinkBeforeShrink. This repository also contains a synthetic copy of the Helius data and an example script to guide users.

## 6. Discussion

We observed that grouping of covariates to allow for differential penalties, that is, group-adaptive shrinkage, may improve predictive performance. Here, we only studied the low-dimensional linear regression setting, but we have observed similar results for the high-dimensional logistic ridge setting.^21^ One might argue that the grouping of covariates is subjective. First, this is not the case when the set of covariates presents a clear grouping structure. Two examples are dummies that correspond to the same nominal covariate, which form one group, and a group of biomarkers that is jointly measured representing similar “items” like lipids. Second, subjectivity is alleviated by substantiating the covariate groups through prior knowledge, for example, by simply splitting the set of covariates into two groups: one for which there is substantial evidence of association with the outcome, and one for which this evidence is absent. Moreover, in the latter case, differential shrinkage is much less subjective than the commonly used strategy to simply discard many covariates a priori (for which supposedly little external evidence exists), because this basically assigns an infinite penalty to the excluded covariates a priori. We believe it to be more objective to leave those in, but allow differential shrinkage for those as compared to the others. Finally, we demonstrated that the results are robust against misspecification of the covariate groups.

If calibration is key, global shrinkage methods with flexible penalties, both frequentist and Bayesian ones, are usually not very suitable. This is not surprising as these penalty parameter(s) adapt to predict optimally in terms of accuracy, which is a different goal than calibration. We showed that Bayesian local shrinkage is a good alternative which competes with conventional methods that do not tune penalties, such as OLS, ML, Firth, and ridge with a fixed penalty. This is in line with earlier results,^9^ where the benefit of Bayesian local shrinkage for calibration is demonstrated in a genetic prediction setting. Note that the importance of having calibrated predictions depends on (a) the possibility of recalibrating predictions in a later stage; and (b) the need or desire to interpret the predictions on an absolute scale. For the latter, the design of the study is also very relevant; often recalibration will be needed anyhow when applying the predictor to a population with somewhat different characteristics than the one represented by the study, a very common case for medical studies.

While the grouping of covariates can aid in improving prediction accuracy, it may deteriorate coverage of the confidence intervals of predictions in the classical setting. Here, the additional regularization by the $C^{+}(0,1)$ prior as invoked by the Bayesian procedures helps to improve coverage, while maintaining competitiveness on prediction accuracy and calibration. The importance of quantifying uncertainty of the predictions differs from study to study. However, in any study it will be useful to know it, also to assess whether the sample size should be increased to lower this uncertainty to an acceptable level. As we have established appropriate coverage of the credible intervals of the predictions for those procedures, we suggest to use those for this purpose.

We discussed the linear and logistic case, which could be regarded as two extremes: the former being information-rich with unbiased ML (= OLS) estimates, the latter being information-poor with biased ML estimates. This means that in the latter case mild shrinkage is also beneficial for calibration. Moreover, it may be relatively more beneficial to a use a subjective prior (or penalty) in the logistic setting.^2^ We discussed two solutions that performed well in our small simulation study: prior variance equal to 1/2,^12^ or less subjective, a hyperprior for the variance with median equal to 1/2. A good alternative may be to use a historical prior, which is tuned to available data from similar studies.^22,23^ Then, the solution with a hyperprior may be preferable over one that fixes the variance, as the former allows the current study to deviate somewhat more when it would not behave similarly.

For our applications, computational time was not an issue at all: all methods ran within seconds for given data sets. Nevertheless, the classical methods fitted substantially faster than the Bayesian ones, rendering the former an edge for analyzing larger scale studies. Whether this balances against the demonstrated benefit of higher-level shrinkage that Bayesian methods can provide, will depend on the main aim(s) of the study (prediction accuracy/calibration/uncertainty quantification). In general, Bayesian methods gain popularity in epidemiological research,^23^ which assists their acceptance for general use. Note that, on purpose, we did not tweak the Bayesian methods in terms of convergence checks or use of other hyper-priors, as these would imply further tuning, rendering the comparison with less flexible methods unfair. In terms of software, we recommend to use either mgcv, or R-stan with the $C^{+}$ priors, as both incorporate estimation of multiple penalty parameters and provide intervals with, in most settings, fairly good coverage. The latter has an edge when calibration is key as it allows for local penalties, or when stabilization of group-adaptive penalties is relevant, for example, for uncertainty quantification. Supplemental Figure 18 shows a flowchart with our recommendations on when to use which method.

Our work is limited in scope, so we discuss several important extensions. The first one is variable selection. As this is a different goal than prediction, other priors and penalties should be discussed. In the classical setting, many variants of the lasso (adaptive, group, and hierarchical) become relevant, and so do (adaptations of) traditional stepwise selection techniques.^24^ It is not trivial, though, to provide appropriate inference for these due to the potential instability of the selection itself. In the Bayesian setting, one may wish to include spike-and-slab priors and/or Zellner’s g-prior, as these are targeting for variable selection.^25^ Alternatively, posterior selection techniques that apply to the fairly dense ridge-type methods used here may be very competitive to those more sparse formulations.^26^

A second extension is to study the effect of shrinkage in the context of causal inference. Many causal inference frameworks make use of prediction methods to account for confounding or non-random treatment allocation. Unlike in predictive modeling, the emphasis in causal inference is primarily placed on bias, and much less so on variance reduction. This makes the use of shrinkage less natural, in particular for the treatment effect. Nevertheless, some (local) shrinkage of the other covariates may be beneficial when p is relatively large compared to n , possibly in combination with double-robust estimation^27^ to counter misspecification of the model.

A third extension is the multi-regression setting, as often encountered in high-dimensional multiple testing setting, for example, when relating gene expression to phenotype, correcting for confounders like age and gender. In such a setting, shrinking effects across similar features (e.g. genes) may improve effect size estimates and multiple testing properties.^28^ Moreover, a multi-regression setting allows tuning of hyper-parameters of the penalty’s prior across features using empirical Bayes.^29^

Sample sizes like n=100,200 are fairly common in medical research. A cautionary note, however, is appropriate: such sample sizes do usually not suffice to establish the final prediction model. Shrinkage is only a partial solution for underpowered studies. In the end, increase of sample size will often be needed to draw firm conclusions. However, we do believe shrinkage may play an important role in the research cycle, which for practical reasons often starts with a small study. Then, as demonstrated, well-thought global or group-adaptive shrinkage methods can help to better assess the predictive potential of the study and quantify uncertainty of the predictions, whereas local shrinkage methods reduce bias and improve calibration. Therefore, these shrinkage methods aid in deciding whether and how to extend the study.

**Recommendations**

1. Specify the focus: Prediction accuracy or calibration, as this determines the most suitable shrinkage method.
2. Local shrinkage (Bay_loc) is recommended for good calibration and better accuracy than OLS and ML.
3. If possible, enlarge the study according to available sample size calculation methods.
4. Linear regression:
  - Global shrinkage (ridge and Bay_glo) is recommended for prediction accuracy, when covariate groups are absent.
  - When k groups of covariates are present, use group-adaptive shrinkage (ridge_k and Bay_k), with a preference for the latter when coverage of confidence intervals is key
5. Logistic regression:
  - Use mild shrinkage (Firth, ridge05, and Bay_loc) when calibration is a major concern, with Bay_loc being less prone to global over- or undershrinkage.

## Supplemental Material

sj-pdf-1-smm-10.1177_09622802251338440 - Supplemental material for Alternatives to default shrinkage methods can improve prediction accuracy, calibration, and coverage: A methods comparison study Supplemental material, sj-pdf-1-smm-10.1177_09622802251338440 for Alternatives to default shrinkage methods can improve prediction accuracy, calibration, and coverage: A methods comparison study by Mark A van de Wiel, Gwenaël GR Leday, MartijnWHeymans, Erik W van Zwet, Ailko H Zwinderman and Jeroen Hoogland in Statistical Methods in Medical Research
