# Supplementary material for: Alternatives to default shrinkage methods can improve prediction accuracy, calibration, and coverage: A methods comparison study
Source: Stat Methods Med Res. 2025 May 29;34(7):1342–55. doi: 10.1177/09622802251338440 (PMC12308036; doi:10.1177/09622802251338440)
Supplement: sj-pdf-1-smm-10.1177_09622802251338440 - Supplemental material for Alternatives to default shrinkage methods can improve prediction accuracy, calibration, and coverage: A methods comparison study [file sj-pdf-1-smm-10.1177_09622802251338440.pdf]

SUPPLEMENTARY MATERIALS TO: Alternatives to default shrinkage methods can improve prediction accuracy, calibration and coverage: A methods comparison study

Mark A. van de Wiel[1], Gwenaël G.R. Leday[2], Jeroen Hoogland[1], Martijn W. Heymans[1], Erik W. van Zwet[3], Ailko H. Zwinderman[1]

[1]*Dept of Epidemiology and Data Science, Amsterdam Public Health research institute, Amsterdam University Medical Centers, Amsterdam, The Netherlands;* [2]*Biometris, Wageningen University and Research, Wageningen, The Netherlands;* [3]*Dept of Biomedical Data Sciences, Leiden University Medical Center, Leiden, The Netherlands*

1 Equivalence of MSEp and PMSE

Define the mean squared error of prediction for a given test set T:

$$\text{MSEp} = 1/|T| \sum_{i \in T} (\eta_i - \hat{\eta}_i)^2,$$

with, for test sample i, true $\eta_i = \beta_0 + X_i\boldsymbol{\beta}$ and estimated $\hat{\eta}_i = \hat{\beta}_0^s + X_i\hat{\boldsymbol{\beta}}$, where $\hat{\beta}_0$ and $\boldsymbol{\beta}$ are estimated from independent training samples. Analogously, define the prediction mean squared error (PMSE) by replacing η_i by response $y_i = \eta_i + \epsilon_i$. Then, in expectation, these two are equal:

$$\begin{aligned} E_y[(y_i - \hat{\eta}_i)^2] &= E_y[((y_i - \eta_i) + (\eta_i - \hat{\eta}_i))^2] = E_\epsilon[(\epsilon_i + (\eta_i - \hat{\eta}_i))^2] \\ &= (\eta_i - \hat{\eta}_i)^2 + E[\epsilon_i^2] + 2E[\epsilon_i](\eta_i - \hat{\eta}_i) = (\eta_i - \hat{\eta}_i)^2 + C, \end{aligned}$$

with constant $C = \sigma^2$.

2 Variability of penalty parameters

The left-hand side of Figure 6 confirms the previously reported instability of the penalty parameter(s) [1] for our data: the penalty estimates may differ 2-3 natural logs in magnitude from one subset to another ($n = 100$), with standard ridge and Bay_glo alike. Hence, the extra regularization of Bay_glo has little effect here. When multiple penalties are estimated, however, extra regularization can improve stability of those, as illustrated in Figure 7: Bay_2 compares favourably to ridge_2 for stability of λ_2. Nevertheless, between subset variability of $\boldsymbol{\lambda}$ remains considerably large. Therefore, we argue that it is important to propagate the uncertainty of $\boldsymbol{\lambda}$ into estimation of $\boldsymbol{\beta}$ when analysing *one* subset, provided that this reflects the *between* subset variability, as the latter is usually not available. The right-hand side of Figure 6 shows this: when using Bay_glo the posterior variability of $\boldsymbol{\lambda}$ for ten random subsets approximates the between subset variability of the ridge penalty (left-hand side) fairly well, in particular in order of magnitude.

3 Synthetic data

Data from the Helius study [2] were used as a running example throughout the manuscript, but they cannot be shared due to privacy regulations. To enhance reproducibility of our results, we provide a synthetic copy of the Helius study data to render qualitatively similar results as compared to the real data. This supplement describes construction of the synthetic data set.

In essence, the problem was approached from a missing data perspective. After applying standardization and the removal of rows with missing data (as described in the main manuscript), the original data set of size $n \times m$ was augmented with an empty data set of size $n \times m$. Subsequently, the empty rows were imputed using imputation by chained equations [3, 4] as implemented in the `mice` package [5] in `R`. This procedure makes use of univariate imputation models for every incomplete variable, and uses an iterative updating scheme that iterates between imputation and updating of the imputations models. The specific application of (multiple) imputation by chained equations for the creation of synthetic data was described by [6].

For this particular application, the variables systolic blood pressure, age, gender, BMI, ethnicity (5 levels), smoking (binary), packyears, coffee (binary), glucose (log), and cholesterol, had to be imputed. Predictive mean matching [4] was used for all continuous variables, logistic regression imputation was used for binary variables, and a multinomial imputation model was used to impute ethnicity. All imputation models were linear additive models conditional on all of the remaining variables. For example, the imputation models for age was a linear additive model with main effects of systolic blood pressure, age, gender, BMI, ethnicity (dummy coded, 4 df), smoking (binary), packyears, coffee (binary), log glucose, and cholesterol level. A single imputed data set was created to represent a synthetic version of the helius data. Traceplots were stable after the chosen number of 25 iterations for the *mice* algorithm. The synthetic data set was found to closely resemble the original data with respect to mean structure, variance, and covariance structure, with a mean absolute deviation from the original data < 0.01 on the standardized data for all three. The synthetic data set did not contain any duplicated rows from the original data. As a further check, a random forest was fitted with the aim to distinguish the original and synthetic data [7, 8]. Half of the data was used for training, and half for testing, and 500 trees were fitted. The test Brier score was 0.23 (with random providing 0.25), indicating that the forest could not accurately separate real and synthetic data.

It is worth noting that the linear additive nature of the imputation models suited our purposes well, but that it does not provide a general solution for synthetic data generations, because it does not reflect possible non-linear relations and interactions. Other possibilities are available, such as multiple imputation using classification regression trees [6]. Also, it should be noted that use of a single imputed data set does not reflect the uncertainty of the imputed (synthetic) values. For synthetic data to be used to replicate inference results, where this uncertainty is key, multiple imputed/synthetic sets would be required [6].

4 Calculation of sample size $n = 320$

To study to what extent differences between shrinkage methods diminish when further increasing sample size, we provide results for $n = 100, 200, 320$. The latter results from the sample

size calculation in [9], which is based on reducing the need for shrinkage by lower bounding a global shrinkage factor. Specifically, formula (9) in [9] implies that n is the smallest integer larger or equal to n_0, with n_0 solving:

$$0.9 = 1 + \frac{p-2}{n_0 \log\left(1 - \frac{R^2_{\text{adj}}(n_0-p-1)+p}{n_0-1}\right)}.$$

Here, 0.9 is the desired shrinkage factor; being close to 1 means little shrinkage. We have $p = 17$, and $R^2_{\text{adj}} = 0.34$, as estimated from the large Master data set.

5 Linear regression setting: Results for $n = 50$

Figures 11 to 13 show the results for the linear regression setting and training sample size $n = 50$. Figures 11, 12 and 13 align with Main Document Figures 2, 4 and 5, respectively. Note: different Y-axis scales are used due to different ranges. Likewise, Table 2 aligns with Main Document Table 2.

6 Supplementary Figures and Tables

Table 1 shows the empirical correlation between non-noise covariates in the Helius master set. Of note, Smoking is coded -1, 1 for "yes", "no", explaining the negative correlation with PackYears.

	Age	Ge	eG	eM	eS	eT	Sm	PY	Co	BMI	Gl	Ch
Age (Ag)	1.00	-0.04	0.01	-0.14	0.14	-0.14	0.04	0.25	-0.24	0.25	0.40	0.30
Gender (Ge)	-0.04	1.00	0.02	0.03	0.01	-0.03	0.16	-0.27	-0.04	0.12	-0.21	-0.00
etnGha (eG)	0.01	0.02	1.00	-0.16	-0.25	-0.15	0.16	-0.18	0.17	0.09	-0.04	0.01
etnMar (eM)	-0.14	0.03	-0.16	1.00	-0.34	-0.20	0.11	-0.12	-0.05	0.04	0.00	-0.13
etnSur (eS)	0.14	0.01	-0.25	-0.34	1.00	-0.32	-0.10	0.03	0.08	0.01	0.08	0.02
etnTur (eT)	-0.14	-0.03	-0.15	-0.20	-0.32	1.00	-0.11	0.07	-0.05	0.12	-0.00	-0.02
Smoking (Sm)	0.04	0.16	0.16	0.11	-0.10	-0.11	1.00	-0.53	0.02	0.11	0.01	-0.01
PackYears (PY)	0.25	-0.27	-0.18	-0.12	0.03	0.07	-0.53	1.00	-0.11	-0.05	0.14	0.11
Coffee (Co)	-0.24	-0.04	0.17	-0.05	0.08	-0.05	0.02	-0.11	1.00	-0.02	-0.07	-0.09
BMI	0.25	0.12	0.09	0.04	0.01	0.12	0.11	-0.05	-0.02	1.00	0.32	0.07
Gluc (Gl)	0.40	-0.21	-0.04	0.00	0.08	-0.00	0.01	0.14	-0.07	0.32	1.00	0.05
Chol (Ch)	0.30	-0.00	0.01	-0.13	0.02	-0.02	-0.01	0.11	-0.09	0.07	0.05	1.00

Table 1: Correlations between non-noise covariates

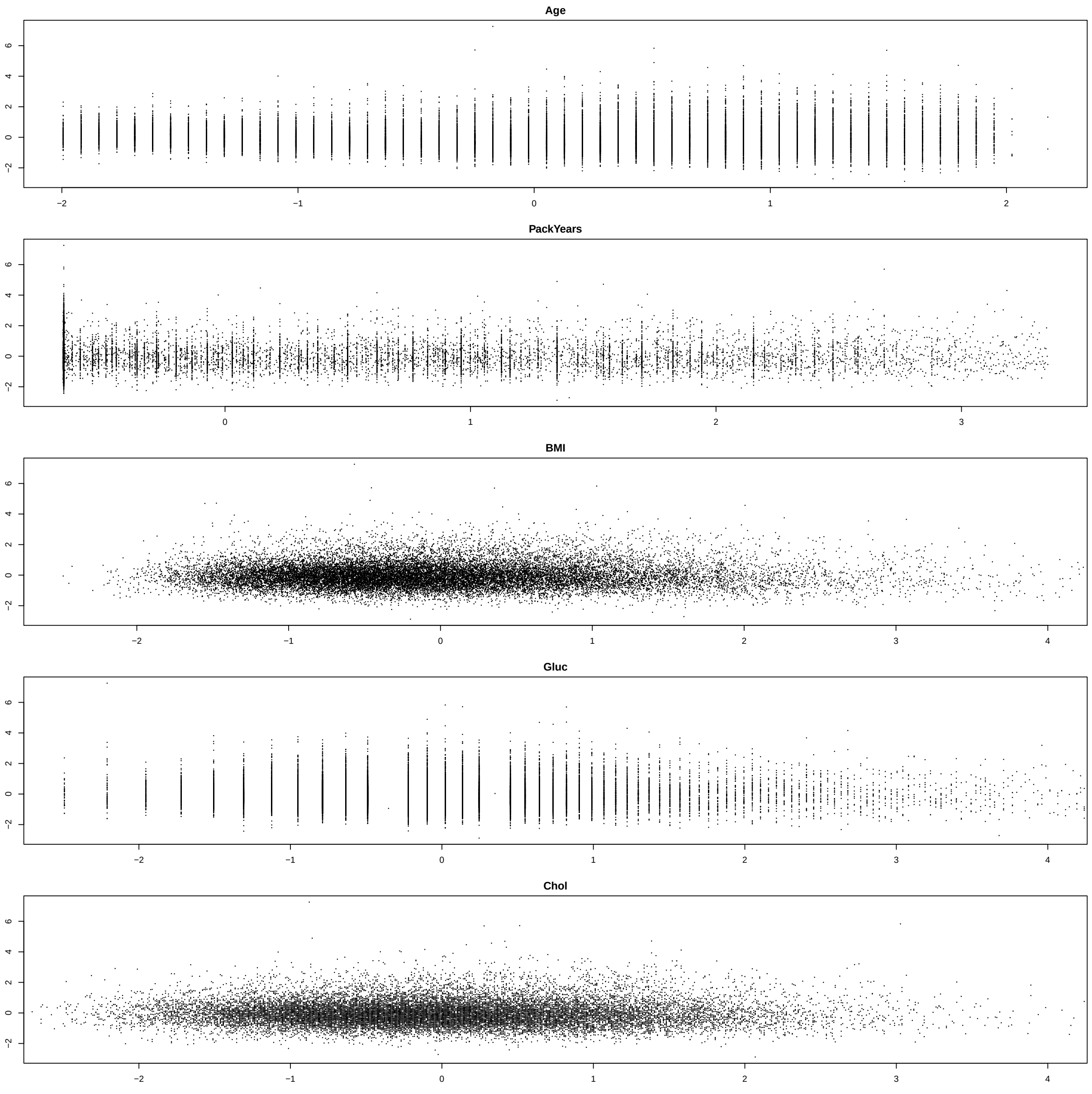

Figure 1: Residual plots for continuous covariates as based on linear regression applied to the entire data set ($N = 21,570$)

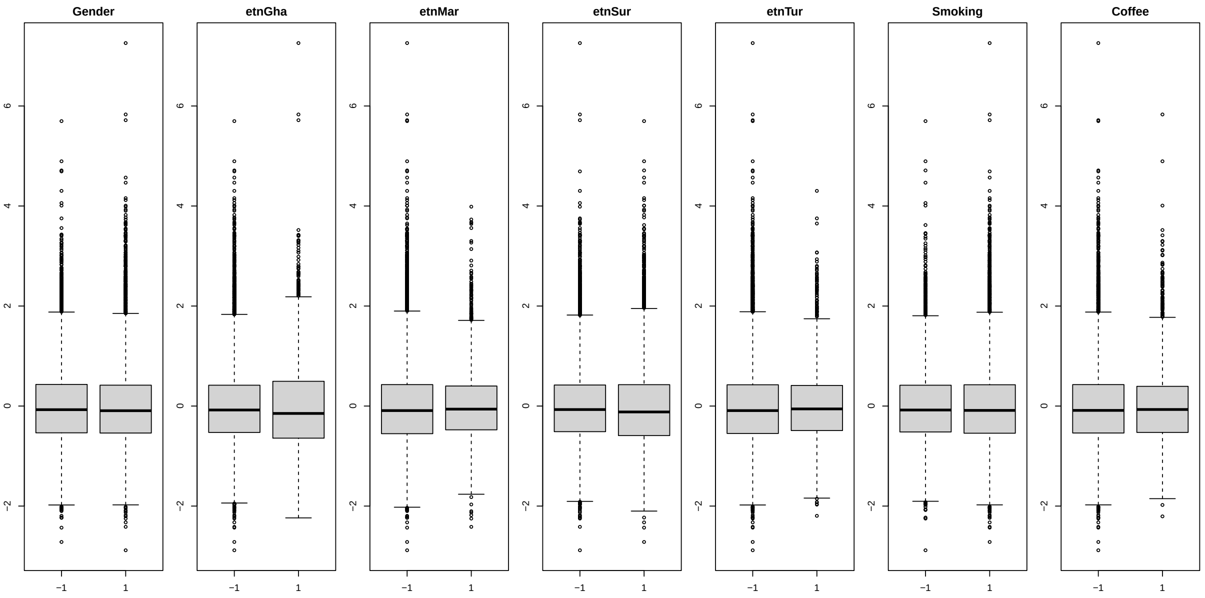

Figure 2: Residual plots for binary covariates as based on linear regression applied to the entire data set ($N = 21,570$)

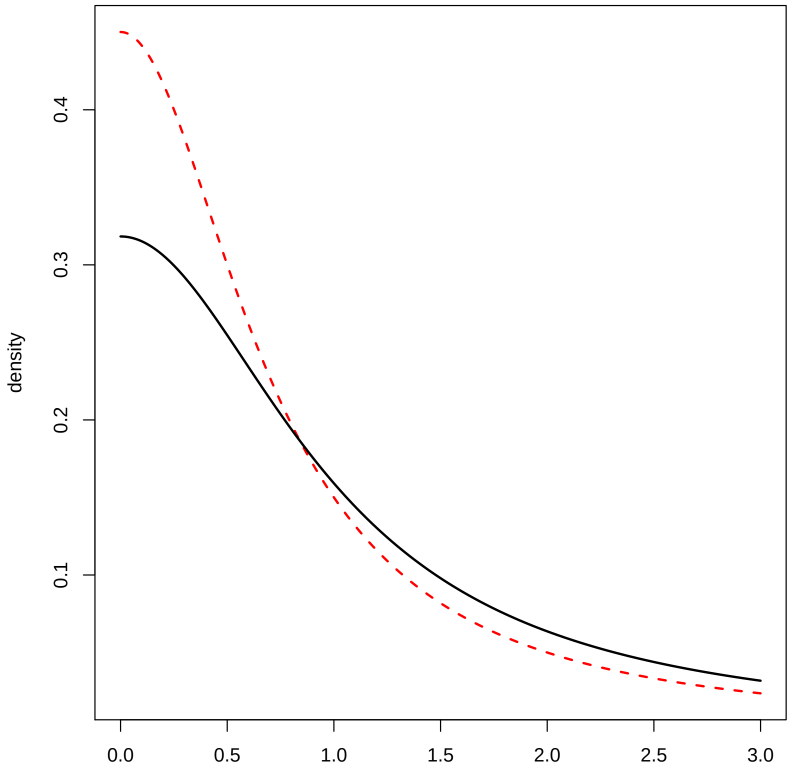

Figure 3: Half-Cauchy priors: $C^+(0,1)$ (solid), $C^+(0,\sqrt{0.5})$ (dashed)

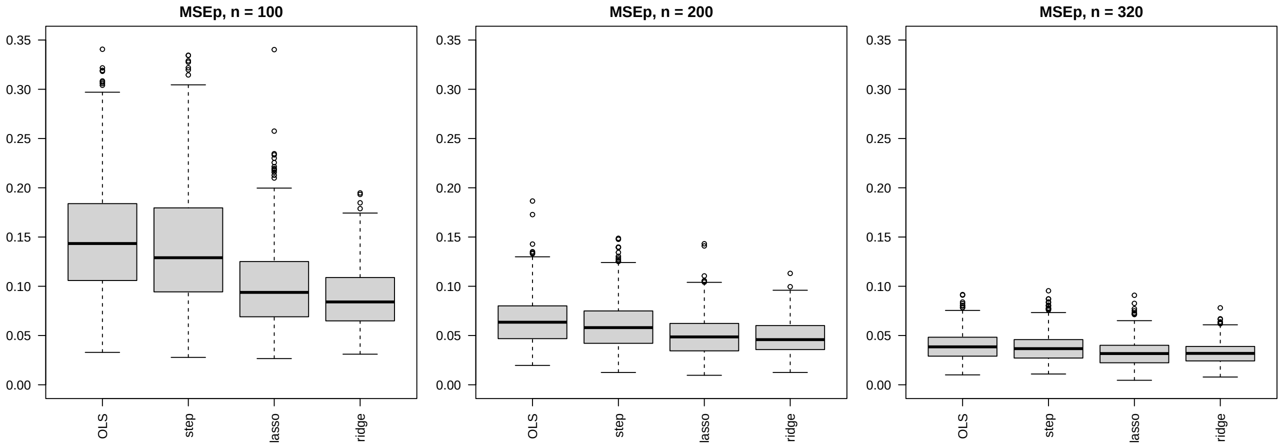

Figure 4: MSEp (y-axis) for several standard methods across 400 subsets

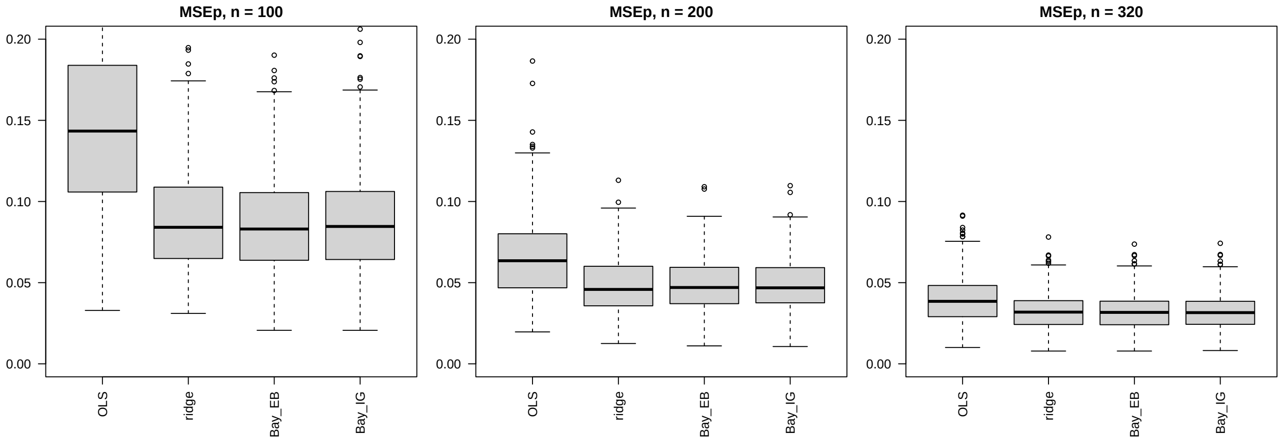

Figure 5: MSEp (y-axis) for standard ridge variants across 400 subsets. Suffices ‘EB’ and ‘IG’ refer to Bayesian ridge with the gaussian variance estimated by empirical Bayes or by full Bayes with an inverse Gamma prior, respectively.

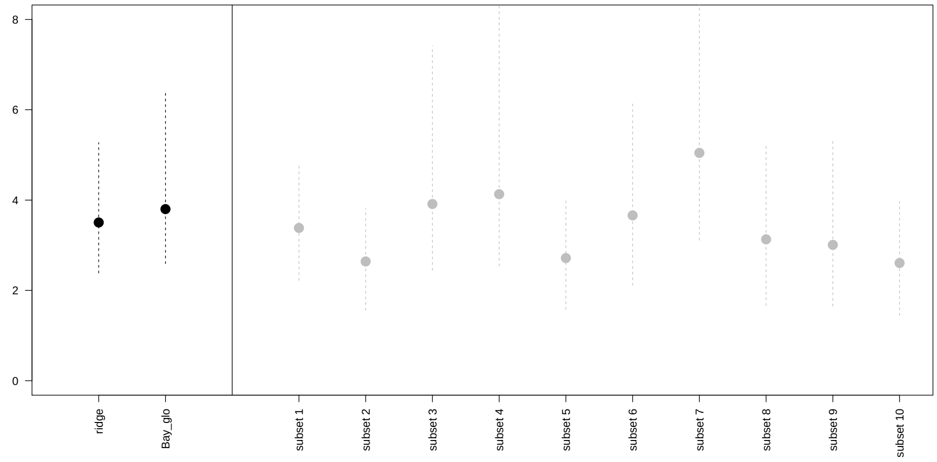

Figure 6: Variability of ridge penalties. Y-axis: ridge penalties on natural log-scale. Left panel (black): ridge penalties estimated by `mgcv` (ridge) and `shrinkage` (Bay_glo; Bayesian ridge with global shrinkage) from *all* 400 subsets; 2.5%, 50% (dot), 97.5% quantiles. Right panel (grey): posterior quantiles of ridge penalty within 10 random subsets using Bay_glo

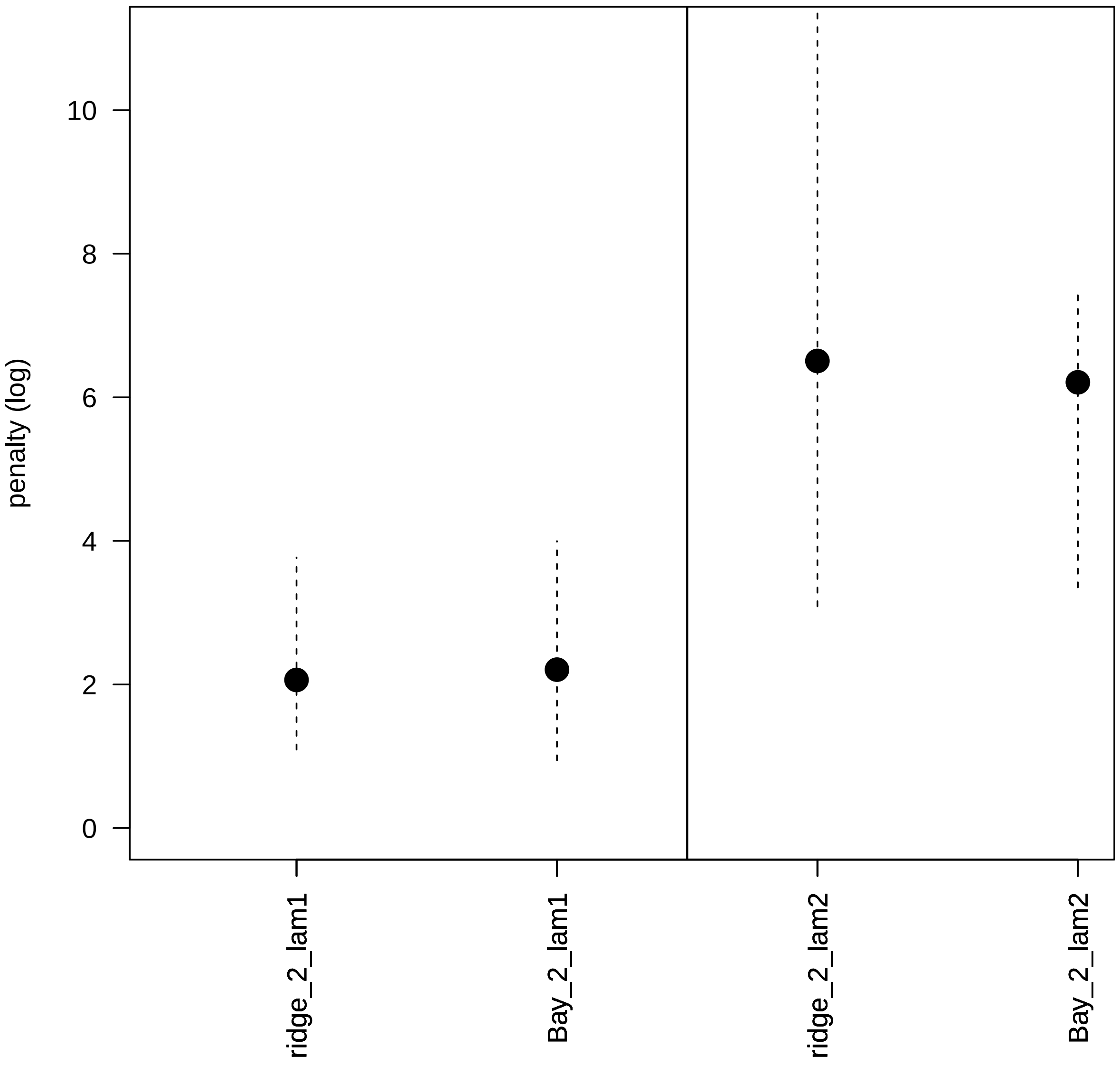

Figure 7: Variability of multi-group (2) ridge penalties. Y-axis: ridge penalties on natural log-scale. Ridge penalties (lam1, lam2) estimated by `mgcv` (ridge_2) and `shrinkage` (Bay_2) from all 400 subsets: 2.5%, 50% (dot), 97.5% quantiles

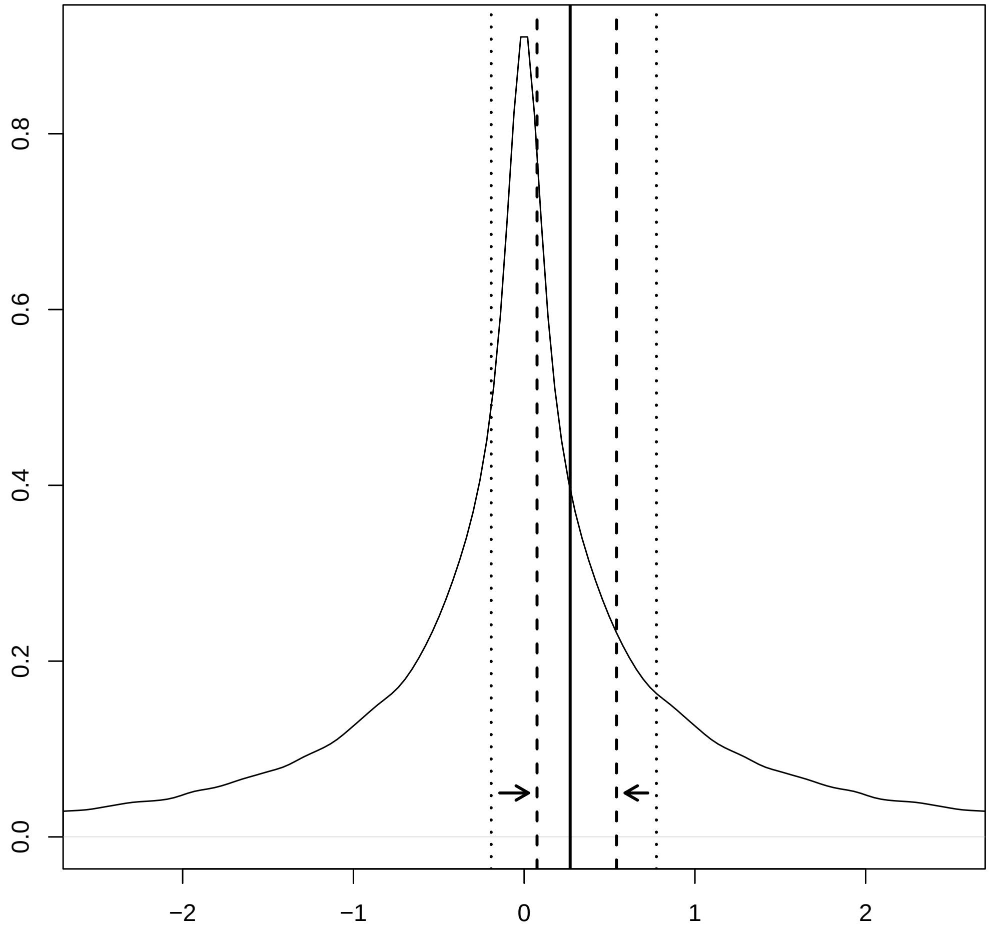

Figure 8: Normal-$C^+(0, 1)$ prior and estimates of β_{BMI} for two subsets of size $n = 50$. Vertical lines: true value of β_{BMI} (solid), OLS estimates (dotted) and Bay_loc (Bayesian local shrinkage) estimates (dashed). Arrows indicate direction of shrinkage by Bay_loc.

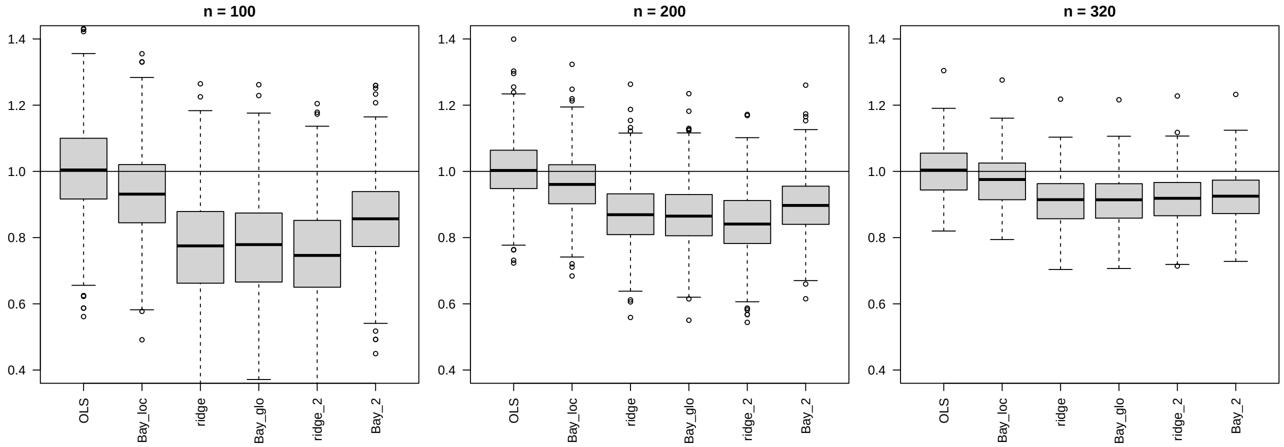

Figure 9: Regression slopes: estimated predictor (used as outcome) against true predictor (used a covariate), across 400 subsets. Suffices 'loc' and 'glo' refer to local and global shrinkage. Digital suffix denotes number of covariate groups for grouped shrinkage.

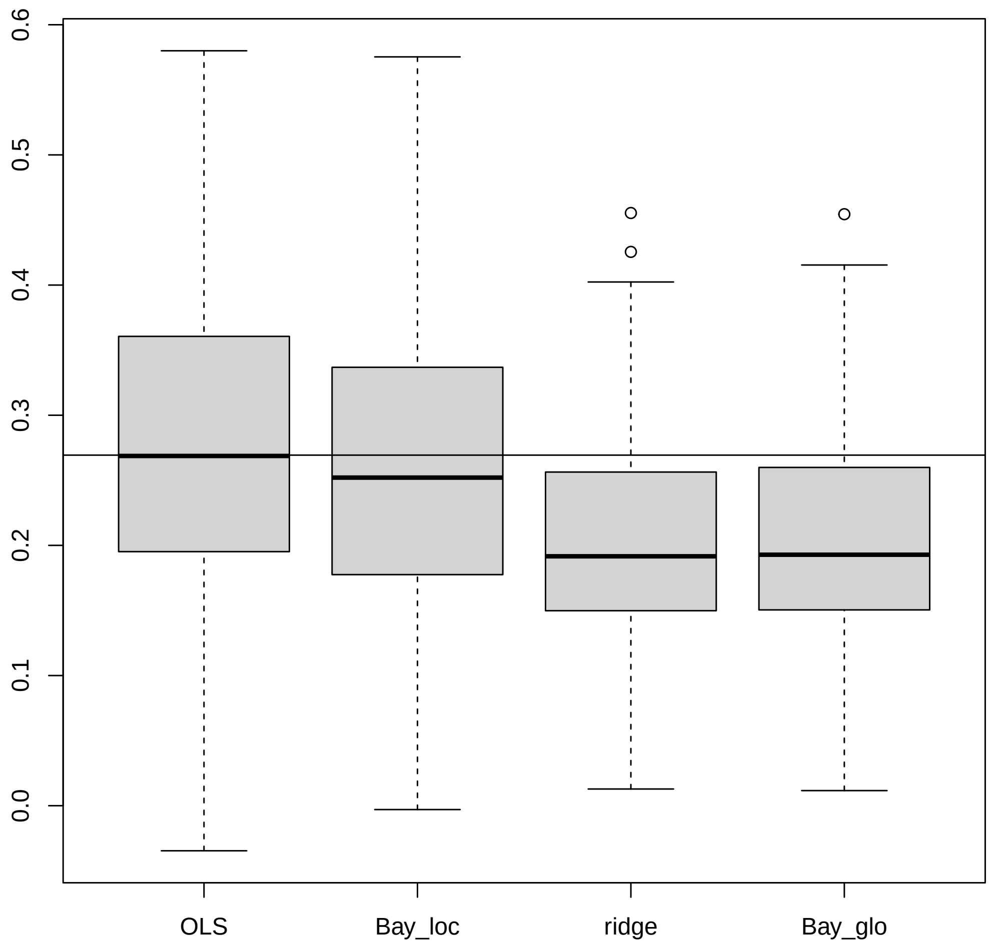

Figure 10: Estimates of β_{BMI} for subsets of size $n = 100$. Horizontal line: true value of β_{BMI}. Suffices ‘loc’ and ‘glo’ refer to local and global shrinkage.

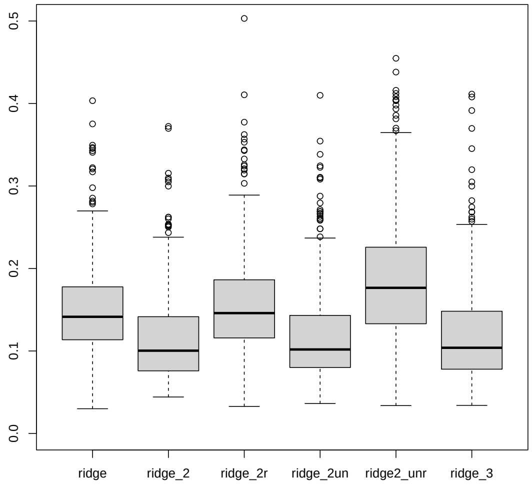

Figure 11: MSEp (y-axis) for group-adaptive ridge penalization across 400 subsets with $n = 50$. Digital suffix denotes number of covariate groups, suffix 'un' denotes one unpenalized covariate group, suffix 'r' denotes random covariate groups

	Coverage		Width	
Methods	Classical	Bayes	Classical	Bayes
OLS, Bay_loc	0.939	0.971	2.334	2.149
ridge, Bay_glo	0.944	0.961	1.521	1.607
ridge_2, Bay_2	0.896	0.966	1.216	1.470

Table 2: Mean coverage (target: 0.95) and width of confidence intervals of predictions for $n = 50$ using classical and Bayesian methods. Suffices 'loc' and 'glo' refer to local and global shrinkage. Digital suffix denotes number of covariate groups for group-adaptive shrinkage.

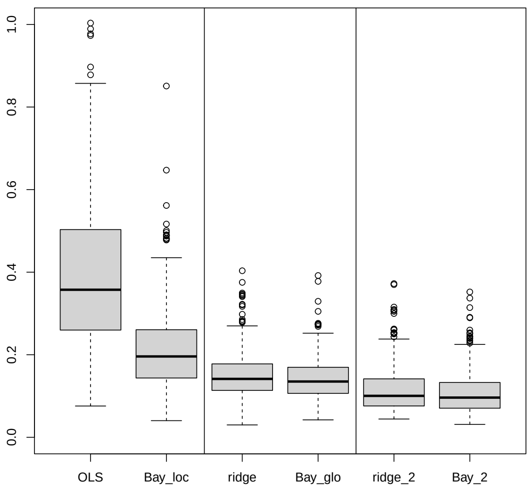

Figure 12: MSEp (y-axis) for classical methods and their Bayesian counterpart across 400 subsets with $n = 50$. Suffices 'loc' and 'glo' refer to local and global shrinkage. Digital suffix denotes number of covariate groups for group-adaptive shrinkage.

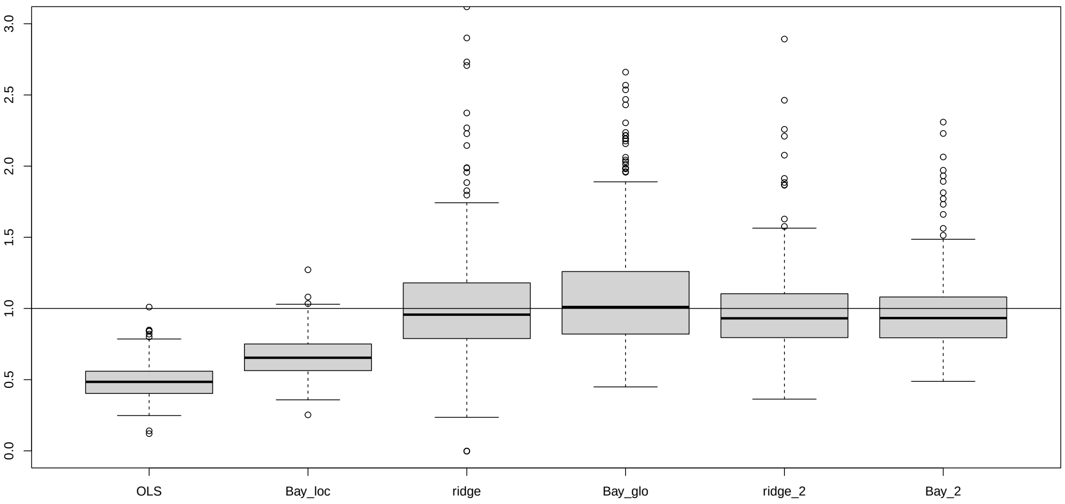

Figure 13: Calibration slopes (y-axis) across 400 subsets with $n = 50$. Suffices 'loc' and 'glo' refer to local and global shrinkage. Digital suffix denotes number of covariate groups for group-adaptive shrinkage

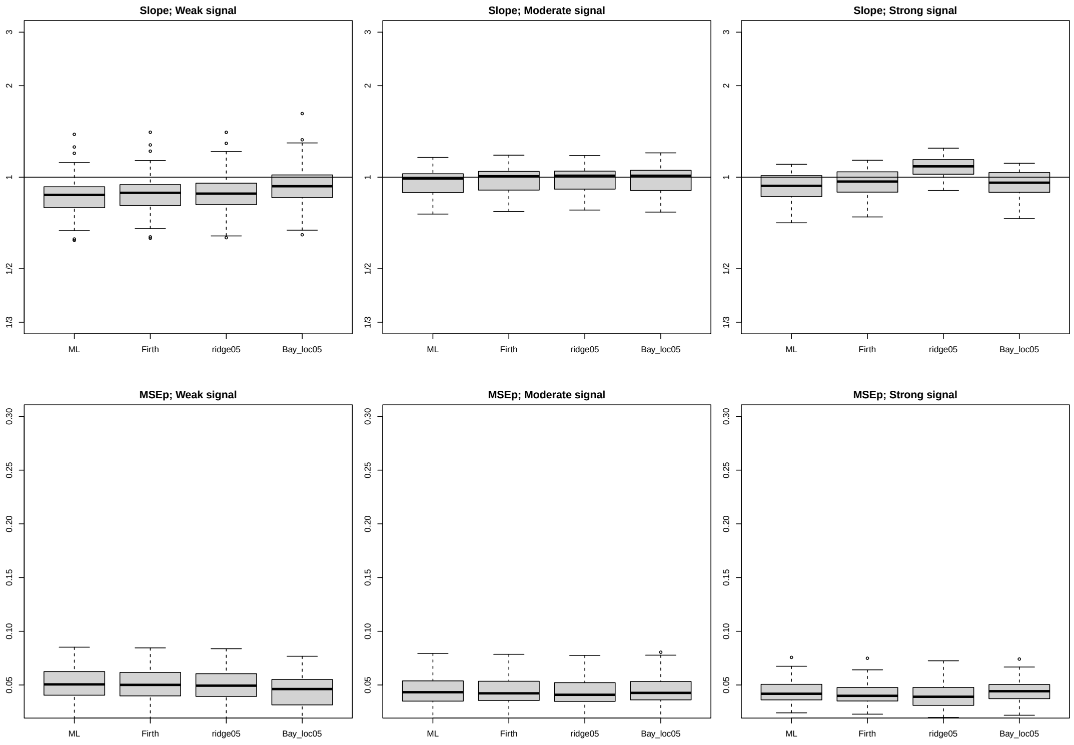

Figure 14: Calibration slope (top, log-scale) and prediction accuracy (bottom; MSEp) for logistic regression simulations, $n = 500$. Simulation setting as described in the main document. Figure on the same scale as for $n = 100$ (main document), to facilitate comparison. Suffix '05' refers to a prior variance or reciprocal ridge penalty equal to 0.5; Suffix 'loc' refers to local shrinkage.

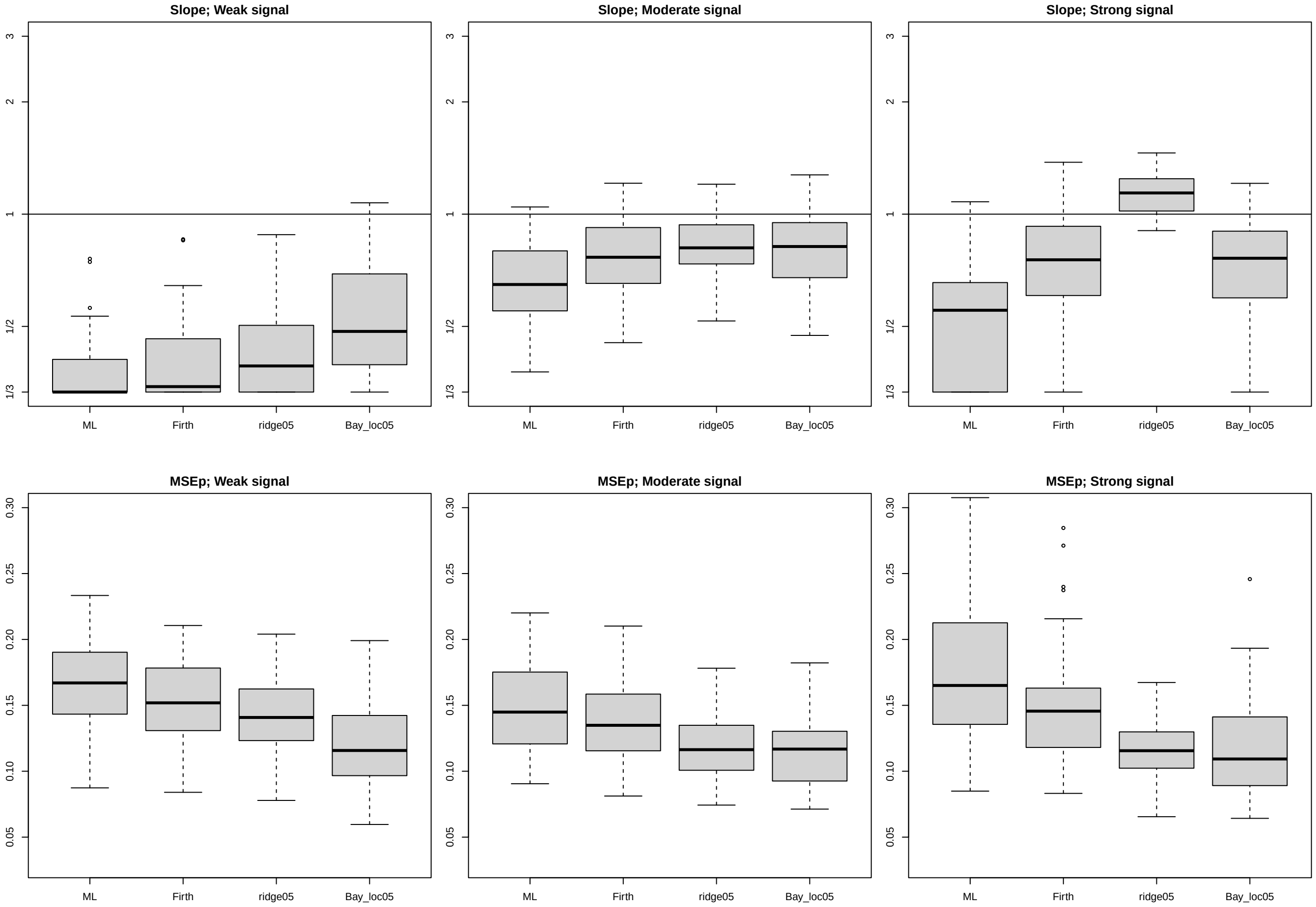

Figure 15: Calibration slope (top; winsorized at $(1/3, 3)$, log-scale) and prediction accuracy (bottom; MSEp) for logistic regression simulations, $n = 100$. Simulation setting as described, but with five β_j's added for which $\beta_j = 0$. Suffix '05' refers to a prior variance or reciprocal ridge penalty equal to 0.5; Suffix 'loc' refers to local shrinkage.

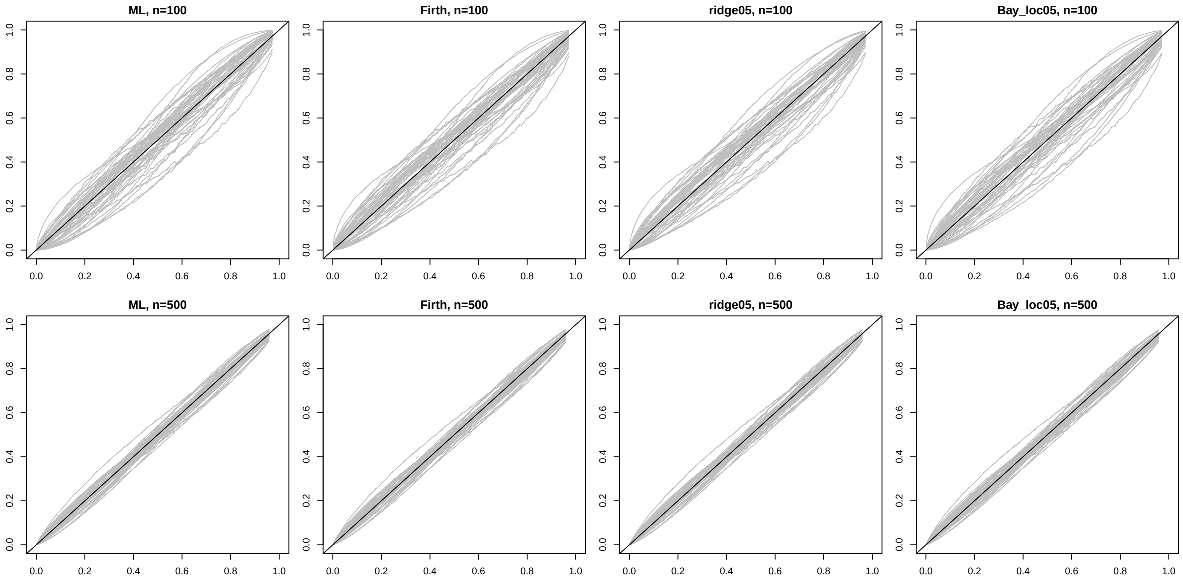

Figure 16: Calibration curves for logistic regression simulations, $n = 100$ (top row) and $n = 500$ (bottom row). Y-axis: ordered predicted test set probabilities, with $n_{\text{test}} = 10^5$. X-axis: true test set probabilities, i.e. fraction of events among 100 neighbouring test samples. Suffix '05' refers to a prior variance or reciprocal ridge penalty equal to 0.5; Suffix 'loc' refers to local shrinkage.

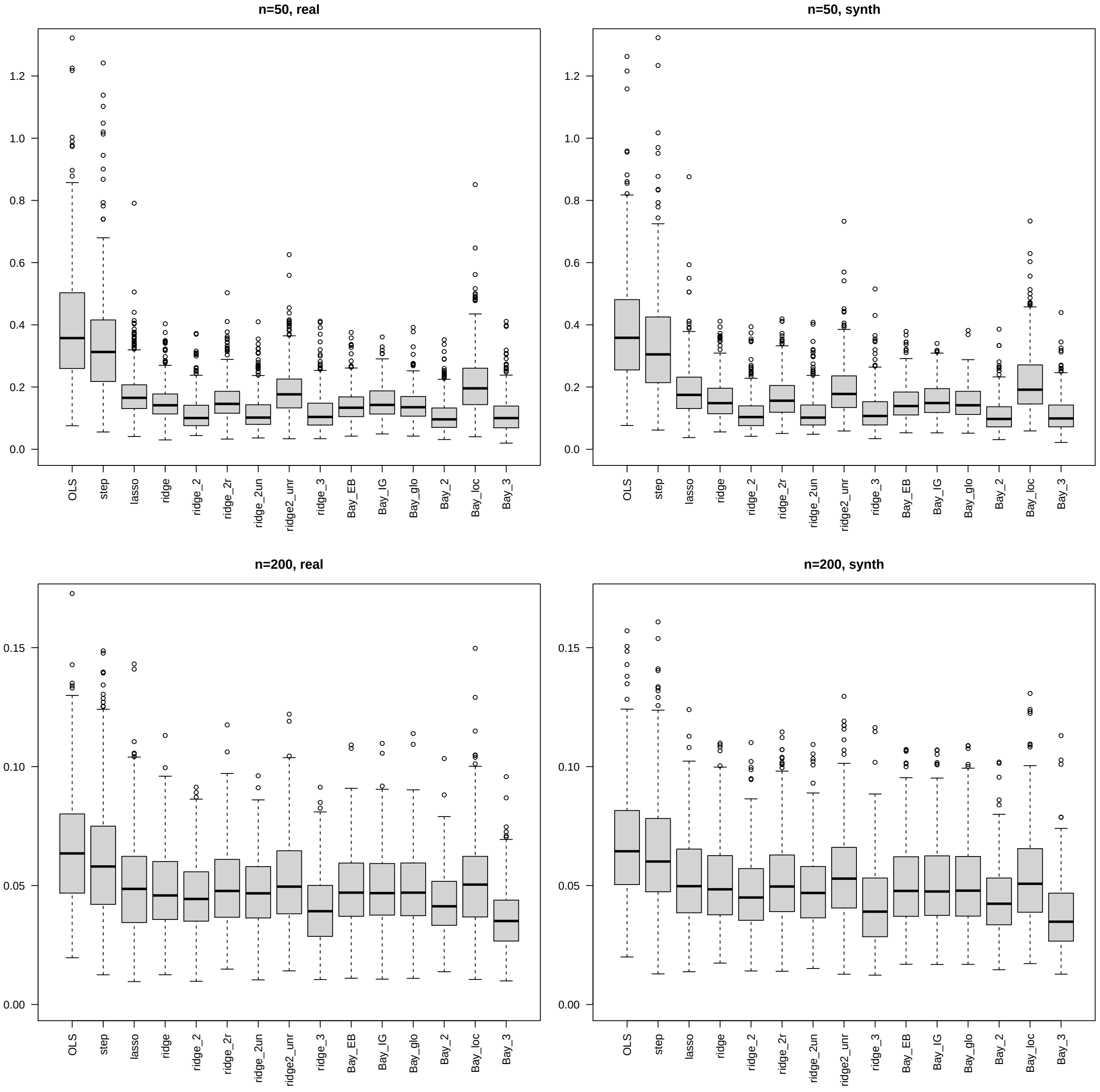

Figure 17: MSE of the predictions for real (left) and synthetic (right) data. Upper row: $n = 50$, bottom row: $n = 200$. Suffices 'loc' and 'glo' refer to local and global shrinkage. Digital suffix denotes number of covariate groups for grouped shrinkage. Suffix 'un' denotes one unpenalized covariate group, suffix 'r' denotes random covariate groups.

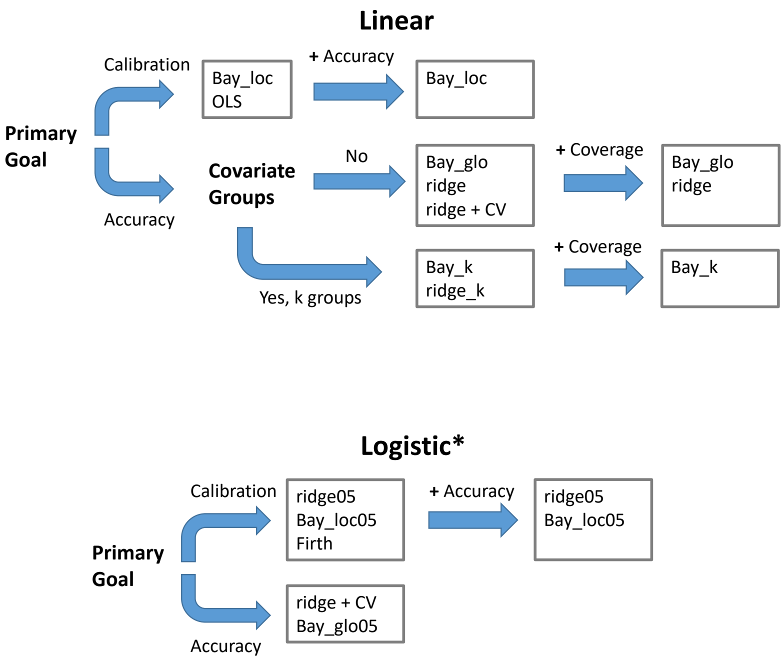

Method	Explanation
ridge05	ridge + fixed penalty = 1/0.5
ridge + CV	ridge + cross-validated penalty (glmnet)
ridge	ridge + uncertainty propagation of penalty, estimated by max marg likelihood (mgcv)
ridge_k	As ridge, but with k penalties
Firth	Logistic regression with Firth correction
OLS	Ordinary least squares
Bay_loc05	Bayesian ridge + local penalties shrunken by half-Cauchy prior with median = 0.5
Bay_glo05	Bayesian ridge + global penalty shrunken by half-Cauchy prior with median = 0.5
Bay_loc	As Bay_loc05, but with median = 1
Bay_glo	As Bay_glo05, but with median = 1
Bay_k	As Bay_glo, but with k penalties

Figure 18: Flow chart for recommended use of various methods, depending on primary goal (Calibration / Accuracy) and secondary goal (+ Accuracy / + Coverage).
*Coverage and effect of covariate groups have not been studied for the logistic setting, so these are not included in this flow chart

References

[1] Richard D Riley, Kym IE Snell, Glen P Martin, Rebecca Whittle, Lucinda Archer, Matthew Sperrin, and Gary S Collins. Penalization and shrinkage methods produced unreliable clinical prediction models especially when sample size was small. *J Clin Epidemiol*, 132:88–96, 2021.

[2] Karien Stronks, Marieke B Snijder, Ron JG Peters, Maria Prins, Aart H Schene, and Aeilko H Zwinderman. Unravelling the impact of ethnicity on health in Europe: the HELIUS study. *BMC Public Health*, 13(1):402, 2013.

[3] Ian R. White, Patrick Royston, and Angela M. Wood. Multiple imputation using chained equations: Issues and guidance for practice. *Statistics in Medicine*, 30(4):377–399, 2011.

[4] Stef van Buuren. *Flexible imputation of missing data*. Chapman and Hall/CRC interdisciplinary statistics series. CRC Press, Taylor & Francis Group, Boca Raton, second edition, 2018.

[5] Stef van Buuren and Karin Groothuis-Oudshoorn. mice : Multivariate Imputation by Chained Equations in R. *Journal of Statistical Software*, 45(3):1–67, 2011.

[6] Thom Benjamin Volker and Gerko Vink. Anonymiced Shareable Data: Using mice to Create and Analyze Multiply Imputed Synthetic Datasets. *Psych*, 3(4):703–716, 2021.

[7] Leo Breiman. Random Forests. *Machine Learning*, 45(1):5–32, 2001.

[8] Andy Liaw and Matthew Wiener. Classification and Regression by randomForest. *R News*, 2(3):18–22, 2002.

[9] Richard D Riley, Kym IE Snell, Joie Ensor, Danielle L Burke, Frank E Harrell Jr, Karel GM Moons, and Gary S Collins. Minimum sample size for developing a multivariable prediction model: Part i–continuous outcomes. *Statistics in medicine*, 38(7):1262–1275, 2019.
